# The Double Life of Plant-Based Food Waste: A Source of Phenolic Acids and a Carrier for Immobilization of Lipases Capable of Their Lipophilization

**DOI:** 10.3390/ijms262311400

**Published:** 2025-11-25

**Authors:** Karina Jasińska, Bartłomiej Zieniuk, Marcin Bryła, Daria Padewska, Rita Brzezińska, Bartosz Kruszewski, Dorota Nowak, Agata Fabiszewska

**Affiliations:** 1 Department of Chemistry, Institute of Food Sciences, Warsaw University of Life Sciences (WULS-SGGW), 159c Nowoursynowska Street, 02-776 Warsaw, Poland; bartlomiej_zieniuk@sggw.edu.pl (B.Z.); rita_glowacka@sggw.edu.pl (R.B.); 2 Department of Food Safety and Chemical Analysis, Waclaw Dabrowski Institute of Agricultural and Food Biotechnology—State Research Institute, Rakowiecka 36, 02-532 Warsaw, Poland; marcin.bryla@ibprs.pl (M.B.); daria.padewska@ibprs.pl (D.P.); 3 Department of Food Technology and Assessment, Institute of Food Sciences, Warsaw University of Life Sciences (WULS-SGGW), 159c Nowoursynowska Street, 02-776 Warsaw, Poland; bartosz_kruszewski@sggw.edu.pl; 4 Department of Food Engineering and Process Management, Institute of Food Sciences, Warsaw University of Life Sciences (WULS-SGGW), 159c Nowoursynowska Street, 02-776 Warsaw, Poland; dorota_nowak@sggw.edu.pl

**Keywords:** food waste, lipase immobilization, chlorogenic acid, enzymatic lipophilization, bioactive compounds

## Abstract

Addressing global food waste challenges, this study investigated plant-based byproducts, spent coffee grounds, apple, and chokeberry pomaces, as sources of phenolic acids and biodegradable carriers for lipase immobilization. The goal was to enhance the lipophilicity and functionality of natural phenolics by enzymatic lipophilization. Microbial lipase from *A. oryzae* was immobilized on these materials, with native spent coffee grounds (NSCG) showing the highest activity (6.0 U/g hydrolytic; 1036 U/g synthetic). Chlorogenic acid (CGA), predominant in extracts, served as a model substrate. Using response-surface methodology, optimal conditions for butyl-CGA synthesis were determined. This is the first report of CGA lipophilization using food-waste-immobilized biocatalysts, where reaction yield for NSCG increased with alcohol chain length, peaking with dodecanol (34.06%). Among synthesized esters, butyl chlorogenate displayed the highest antioxidant activity, comparable to free CGA and BHT, and increased lipophilicity, though a “cut-off” effect appeared for longer chains. Medium-chain esters (C6, C8) showed selective antimicrobial activity against Gram-positive bacteria. While lipophilization of chokeberry pomace and spent coffee grounds extracts reduced antioxidant activity, short-chain esters (C4–C6) improved rapeseed oil stability. The findings highlight food waste as a sustainable source for developing biocatalysts and value-added bioactives with enhanced functional properties.

## 1. Introduction

Food waste is a significant global issue that affects environmental, social, and economic concerns. According to the Food Waste Index Report [1], approximately 931 million tons of food waste were generated in 2019. Of this total, 61% originated from households, 26% from the food service sector, and 13% from retail. This suggests that approximately 17% of the total global food production may be wasted. A study conducted by Poore and Nemecek [2] revealed that nearly one-quarter (24%) of the emissions associated with food production result from losses in supply chains or waste generated by consumers. Specifically, 15% of these emissions are attributed to issues in the supply chain, including inadequate storage, improper handling methods, insufficient refrigeration, and spoilage during transportation and processing. The remaining 9% stems from food discarded by retailers and consumers. Consequently, food waste accounts for approximately 6% of the total global greenhouse gas emissions. The two main groups based on food loss are roots, tubers, and oil-bearing crops (26%) and fruits and vegetables (22%). These include peels, stems, seeds, shells, bran, germs, culls, pomace, pulp, and other residues from processing [3]. These byproducts represent significant sources of bioactive molecules with a wide range of applications. Food waste can be a source of polyphenols, dietary fiber, carbohydrates, and proteins. These molecules have potential for use in functional foods, nutraceuticals, pharmaceuticals, and beauty care products [4].

One of the groups of bioactive compounds of great interest is natural phenolics, which are known for their antioxidant, antimicrobial, anti-inflammatory, and anticarcinogenic properties [5]. However, their hydrophilic nature limits their biological functions. To enhance their effectiveness, a lipophilic group can be added to their structure, resulting in compounds known as lipo-phenolics. This modification changes the hydrophilic-lipophilic balance, enabling the development of new functionalized bioactive molecules with improved properties compared to traditional hydrophilic phenolics. In detail, lipophilization increases the solubility of phenolics in nonpolar environments and alters the positioning of these compounds within emulsions, thereby potentially enhancing their antioxidant capabilities. In emulsified systems, lipo-phenolics typically position themselves at the interface between the lipid and aqueous phases, providing better protection for fats and oils. As a result, lipo-phenolics have demonstrated remarkable antioxidant properties in both food and cosmetic applications [6,7,8,9]. There are two methods for carrying out lipophilization: chemical and enzymatic. The chemical method is more complex due to the thermal sensitivity and oxidation susceptibility of phenolic acids in alkaline media. It involves multiple purification steps and generates waste. In contrast, the enzymatic method is preferred, as it operates under milder conditions, making it an environmentally friendly process that produces fewer byproducts [10,11,12].

Lipases, known as triacylglycerol hydrolases (EC 3.1.1.3), are effective biocatalysts for modifying phenolic compounds. Their primary function is to catalyze the hydrolysis of ester bonds at the interface between hydrophilic and hydrophobic environments. Under specific conditions, particularly in the absence of water, lipases can also catalyze synthesis reactions, such as esterification and transesterification [13,14]. These enzymes find broad applications across various fields, including biotechnology, pharmacy, biodiesel production, bioremediation, detergents, and the food industry, primarily due to their chemo-, regio-, and stereoselectivity. Lipase can be obtained from various sources, including animal, plant, or microbial origins. The last one started to show promise due to its low manufacturing cost and greater availability compared to animal and plant lipases [15].

The use of free enzymes in industry presents several challenges, including low stability under extreme conditions. To address these issues, the immobilization process can be employed. This technique enhances the catalytic activity of enzymes, increases their stability across a broad range of pH levels and temperatures, and facilitates easy recovery and reuse from the reaction mixture [16]. Immobilization can be categorized into two types of interactions: physical and chemical. Physical interactions include hydrogen bonds, van der Waals forces, and hydrophobic interactions, while chemical interactions involve covalent bonding. The primary methods of immobilization include physical adsorption, entrapment or encapsulation, covalent binding, and cross-linking. Various materials serve as carriers for enzyme immobilization. Natural polymers such as gelatin, cellulose, chitin, chitosan, and agarose, as well as nanostructured materials like metal–organic frameworks (MOFs), nanoparticles, carbon nanotubes, and graphene oxide, have already been utilized [17,18,19].

One of the most recognized enzyme preparations is Novozym 435, which is a lipase B from *Candida antarctica* (CALB) immobilized onto macroporous acrylic polymer resin—Lewatit VP OC 1600. This enzyme performs exceptionally well in organic synthesis and biocatalysis [15,20]. Nowadays, new, more biodegradable carriers for enzyme immobilization are being searched for, which would be as effective biocatalysts as the Lewatit adsorbed one. The increasing interest in sustainable practices has heightened efforts to develop effective strategies for repurposing lignocellulosic materials, including apple and chokeberry waste generated during the production of juice and jam. Waste materials from the fruit and vegetable industry, which possess appropriate porosity, surface charge, and chemical inertness, can serve as efficient carriers in enzyme immobilization processes. Over the years, various by-products have been evaluated as potential supports for immobilization, such as brewer’s spent grain, spent coffee grounds, cashew apple waste, sugarcane bagasse, and coconut residues [21,22,23,24,25,26,27,28]. Several of these materials have shown improved enzyme yields. Notably, spent coffee grounds (SCG) have received significant attention, as their global annual generation reaches approximately 6 million tons, accounting for up to 60% of processed coffee beans. With the continuous rise in global coffee consumption, the volume of SCG waste is expected to increase further. SCGs are an inexpensive, renewable lignocellulosic feedstock with desirable properties for enzyme immobilization, such as mechanical strength, chemical inertness, and structural stability. Their composition, rich in organic compounds such as fatty acids, lignin, cellulose, hemicellulose, and other polysaccharides, makes them a valuable matrix for biocatalyst support [29,30,31]. Despite these well-documented characteristics, SCG has rarely been employed as an enzyme carrier in practice. Literature reports on their use have been limited to the immobilization of lipase [24,32], β-glucosidase [33], and cellulase [34].

This study implements a dual-valorization approach for beverage-processing residues: spent coffee grounds, apple pomace, and chokeberry pomace, by using them both as (i) biodegradable supports for immobilizing a microbial lipase and (ii) sources of phenolic acids. We initially developed a chlorogenic acid (CGA) model to optimize enzymatic lipophilization (adjusting temperature, enzyme loading, alcohol: CGA ratio, and reaction time), then applied the optimal conditions to non-purified, polyphenol-rich extracts. To our knowledge, this is the first demonstration of CGA lipophilization with a biocatalyst immobilized onto carriers derived from food waste. By modifying individual phenolics and then whole extracts, we aimed to develop lipophilized preparations with improved functional properties, particularly antioxidant activity, for potential use as clean-label food additives.

## 2. Results

### 2.1. Activity Evaluation of Immobilized Lipase

In the first stage of the study, three different biocatalysts were prepared by immobilizing microbial lipase from *A. oryzae* onto biodegradable carriers. Based on previous observations [35,36] demonstrating that lipases adsorbed onto native residues from beverage production displayed the highest catalytic activity, three untreated agri-food residues were selected as supports: chokeberry pomace, apple pomace, and spent coffee grounds. The lipase was adsorbed directly onto these native supports, yielding three dry-form biocatalysts. To confirm the success of the immobilization process, an elemental analysis was conducted on the biocatalysts obtained, as well as on the raw material used as a support. This analysis focused on the carbon, nitrogen, and sulfur content of the samples, as detailed in Table 1. The samples of spent coffee grounds (SCG) exhibited a relatively high carbon content of 48.41%, a low nitrogen content of 2.23%, and a negligible sulfur content of 0.12%. A similar pattern was observed in chokeberry pomace (ChoP), which contained 50.31% carbon, 1.87% nitrogen, and 0.09% sulfur. In contrast, apple pomace (AP) samples showed lower values: 44.62% carbon, 0.99% nitrogen, and 0.07% sulfur. The results for the elemental content align with existing literature for spent coffee grounds [37], apple pomace [38], and berry pomace [39]. Notably, in all immobilized enzyme preparations, an increase in nitrogen percentage was observed compared to the nitrogen content in the food waste alone. This observation suggests a positive adsorption of proteins onto the carrier.

**Figure 1:**
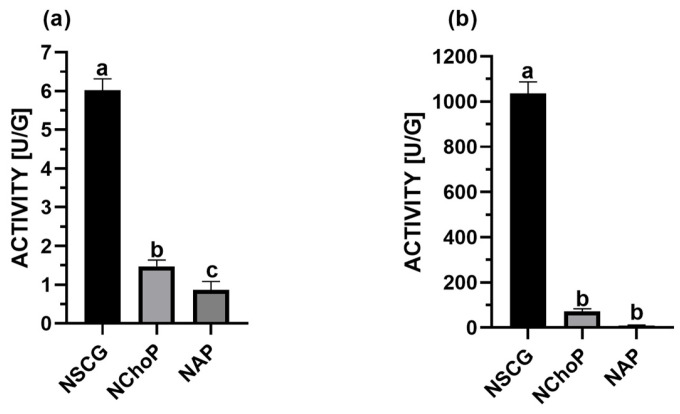
The hydrolytic (**a**) and synthetic (**b**) activities of immobilized lipase onto spent coffee grounds (NSCG), chokeberry pomace (NChoP), and apple pomace (NAP). Means with the same letter (a–c) did not differ significantly (α = 0.05).

The results with hydrolytic and synthetic activity are presented in Figure 1. Among them, the biocatalyst immobilized on spent coffee grounds showed the highest enzymatic activity, with a hydrolytic activity of 6.0 U/g and a synthetic activity of 1036.0 U/g. Significantly outperforming the other two formulations: lipase immobilized on apple and chokeberry pomace. Based on the obtained results, an immobilized biocatalyst onto native spent coffee grounds was selected for further research.

### 2.2. Content of Selected Phenolic Acids in Lyophilized Extracts

**Figure 2:**
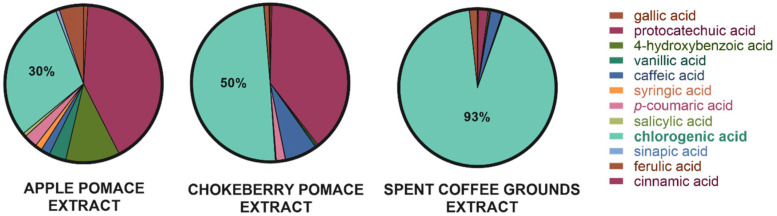
Percentage contribution of selected phenolic acids in freeze-dried extracts from apple pomace, chokeberry pomace, and spent coffee grounds (expressed as % of total identified phenolic acids).

Lyophilized dry extracts obtained from spent coffee grounds, chokeberry pomace, and apple pomace were analyzed for their content of selected phenolic acids using LC-MS (Figure 2). The analysis included the following compounds: gallic acid, protocatechuic acid, 4-hydroxybenzoic acid, vanillic acid, caffeic acid, syringic acid, *p*-coumaric acid, salicylic acid, chlorogenic acid, sinapic acid, ferulic acid, and cinnamic acid. Among the tested samples, the extract derived from spent coffee grounds exhibited the highest dominance of a single compound, with chlorogenic acid accounting for approximately 93% of the total identified phenolic acids. The remaining 7% consisted primarily of protocatechuic, caffeic, and ferulic acids. In the chokeberry pomace extract, chlorogenic acid also represented a significant portion, accounting for approximately 50% of the total phenolic acid content. Protocatechuic and caffeic acids were the next most abundant compounds. The extract from apple pomace displayed a more diverse phenolic profile. In this case, protocatechuic acid was the most prevalent (42%), while chlorogenic acid accounted for approximately 30% of the total, followed by 4-hydroxybenzoic acid as the third most abundant component. Based on the obtained results, chlorogenic acid was selected as a model substrate for further investigations, due to its presence in all three extracts and its relatively high proportion in each sample.

### 2.3. Optimization of Synthesis Reaction

**Figure 3:**
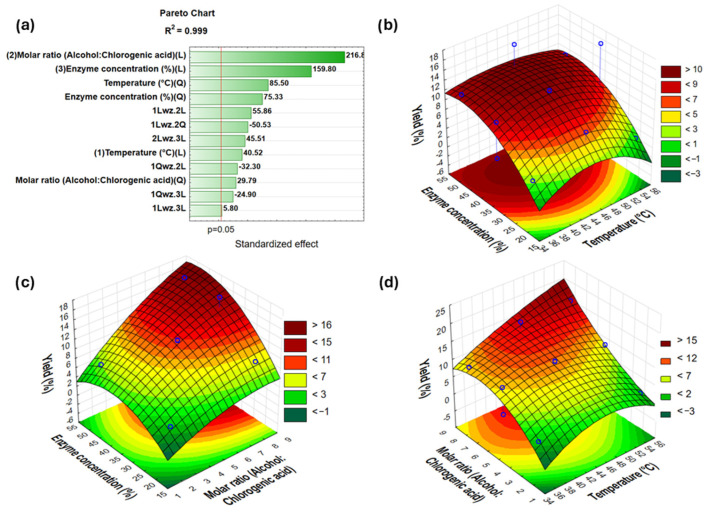
Pareto chart (**a**) and 3D response surface plots showing the effects of the interaction of enzyme concentration and temperature (**b**), enzyme concentration and substrate molar ratio (**c**), and substrate molar ratio and temperature (**d**) on the production yield of butyl chlorogenate catalyzed by NSCG. Explanations: 1—temperature; 2—substrate molar ratio (alcohol: chlorogenic acid); 3—enzyme concentration; L—linear; Q—quadratic; 1Qwz.2L—the interaction between a quadratic effect of temperature and a linear effect of substrate molar ratio in the statistical model, etc.

**Figure 4:**
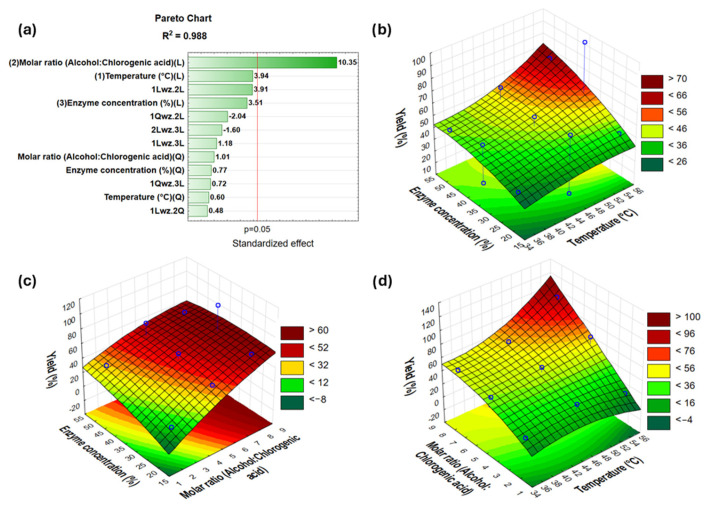
Novozym 435 Pareto chart (**a**) and 3D response surface plots showing the effects of the interaction of enzyme concentration and temperature (**b**), enzyme concentration and substrate molar ratio (**c**) and substrate molar ratio and temperature (**d**) on the production yield of butyl chlorogenate catalyzed by Novozym 435. Explanations: 1—temperature; 2—substrate molar ratio (alcohol: chlorogenic acid); 3—enzyme concentration; L—linear; Q—quadratic; 1Qwz.2L—the interaction between a quadratic effect of temperature and a linear effect of substrate molar ratio in the statistical model, etc.

In the current study, a response surface methodology was utilized to optimize the enzymatic synthesis of butyl chlorogenate via the direct esterification of chlorogenic acid with 1-butanol using NSCG as a biocatalyst and Novozym 435 as a reference. A total of 15 runs (Table 2) were conducted to evaluate different temperatures (35–55 °C), substrate molar ratios (alcohol to chlorogenic acid—2:1, 5:1, and 8:1), and enzyme concentration (20–50%). The results, presented in the form of a Pareto chart and 3D response surface plots, are shown in Figure 3 and Figure 4. The response model evaluated in this study had a coefficient of determination (R^2^) equal to 0.999 and an adjusted R^2^ = 0.999 for reaction catalyzed by NSCG and an R^2^ of 0.988 and an adjusted R^2^ = 0.913 for Novozym 435, with a 95% confidence level.

**Figure 5:**
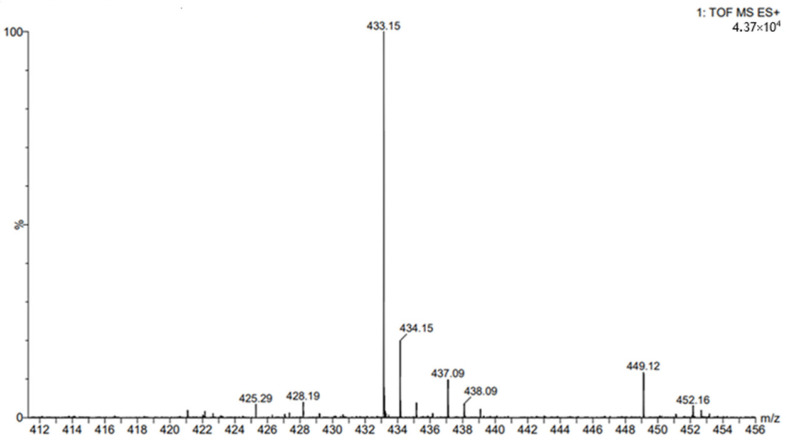
The ESI-MS spectrum (in the selected *m*/*z* range 412–456) for butyl chlorogenate.

The structure of the obtained butyl chlorogenate was confirmed by ESI-MS and NMR analysis. The fragmentation spectrum for the ester derivative of chlorogenic acid is shown in Figure 5.

Based on the Pareto chart for the synthesis of butyl chlorogenate using the NSCG biocatalyst, it was observed that the most significant factors influencing the reaction yield were the linear effect of the substrate molar ratio (1-butanol to chlorogenic acid) and the enzyme concentration relative to the total amount of substrates. The third most influential factor was the quadratic effect of temperature. The corresponding 3D response surface plots confirmed that increasing the alcohol-to-acid ratio led to higher reaction yields. Additionally, both elevated enzyme loading and higher temperatures had a positive impact on the overall efficiency of the esterification process. In contrast, the Pareto chart for the same reaction catalyzed by Novozym 435, used here as a commercial benchmark, showed that only the linear effect of the substrate molar ratio significantly affected the reaction yield; higher alcohol content correlated with improved conversion. Other examined parameters, such as enzyme concentration and temperature, were not statistically significant. This comparison highlights a key distinction between the two biocatalysts: the immobilized lipase on the food waste-derived support (NSCG) exhibited sensitivity to a broader range of reaction conditions, whereas the commercial enzyme immobilized on a synthetic carrier (Novozym 435) demonstrated high robustness and versatility across varying parameters. Nevertheless, following a three-factor Box–Behnken analysis, optimal reaction conditions were established and found to be consistent for both systems. Maximum yields were achieved at 55 °C and at the highest substrate ratio tested, 8:1 (1-butanol: chlorogenic acid). From an economic standpoint, an enzyme concentration of 35% (*w*/*w* relative to total substrates) was selected for subsequent reactions to optimize the process while minimizing biocatalyst usage without compromising efficiency.

**Figure 6:**
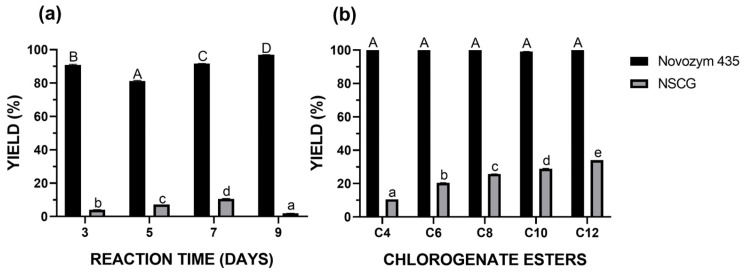
Yield of the reaction for obtaining chlorogenic acid butyl ester depending on the reaction time (**a**) and yield of the reaction for obtaining chlorogenic acid esters with alcohols of varying carbon chain lengths (**b**). The values with the same lowercase letter (a–e) or capital letter (A–D) did not differ significantly (α = 0.05).

**Figure 7:**
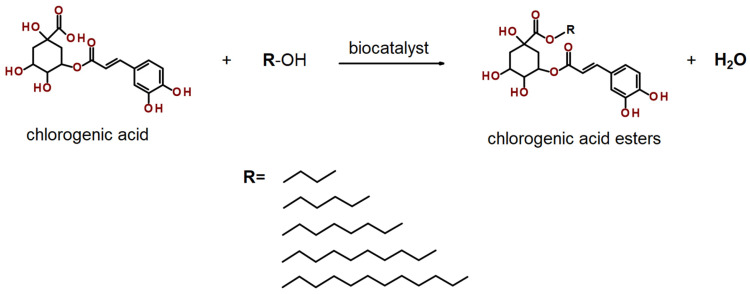
Lipase-catalyzed esterification of chlorogenic acid with butyl, hexyl, octyl, decyl, and dodecyl alcohol.

Further investigations were conducted to examine the influence of reaction time under the established optimal conditions (Figure 6a). For the NSCG biocatalyst, the maximum yield (10.61%) was achieved after 7 days of reaction, whereas Novozym 435 reached its highest yield (97%) after 9 days of reaction. Notably, the commercial immobilized lipase exhibited high catalytic performance early in the process, reaching 90.86% yield after just 3 days, highlighting its superior efficiency in catalyzing this specific esterification. The optimized reaction parameters described above were subsequently applied to lipophilization reactions of chlorogenic acid using a series of fatty alcohols with varying carbon chain lengths (C6, C8, C10, and C12), following the reaction scheme presented in Figure 7. It was observed that for the NSCG biocatalyst, the reaction yield increased proportionally with the length of the alcohol’s carbon chain (Figure 6b). The highest yield (34.06%) was obtained in the reaction with dodecanol (C12), whereas the lowest (10.62%) was recorded with butanol (C4). In contrast, the commercial biocatalyst Novozym 435 showed consistently high yields across all tested alcohols, indicating that the previously optimized reaction conditions were broadly effective for the synthesis of various chlorogenic acid esters using this enzyme. Although the coffee waste-based biocatalyst (NSCG) demonstrated relatively low overall yields, this study represents the first report showcasing its applicability in the enzymatic lipophilization of chlorogenic acid. The results suggest that biodegradable supports derived from food industry residues may serve as a promising, environmentally friendly alternative to conventional immobilization materials in biocatalytic processes.

### 2.4. Extracts Lipophilization

The utilization of whole plant extracts containing diverse phenolic compounds, along with the assessment of their potential synergistic effects, remains a relatively innovative strategy. It is well recognized that extracts derived from different fruit types vary in their phenolic profiles, which directly influence their antioxidant capacities. Consequently, the lipophilization approach must be tailored to the specific composition of each extract [40]. Based on the optimization of butyl chlorogenate synthesis, lipophilization reactions were planned using the obtained lyophilized extracts from food waste. Since chlorogenic acid was identified in all three extracts, the reactions were conducted using the optimal parameters established in the model system. To derivatize chlorogenic acid, 1-butanol was used, with the extract added at a mass ratio of 1:8 (alcohol: extract). An attempt was also made to employ a biodegradable biocatalyst and Novozym 435 as a reference to carry out the lipophilization reaction. The presence of the main reaction product—butyl chlorogenate—was analyzed in these samples to confirm the successful derivatization of chlorogenic acid contained in the extracts. LC-MS analysis confirmed the presence of the chlorogenic acid ester in all reaction mixtures after 7 days of incubation. The conversion rate of butyl chlorogenate is presented in Table 3. The highest conversion rate of butyl chlorogenate was recorded for the lipophilized extract from apple and chokeberry pomace, which reached up to approx. 90%. The prepared NSCG biocatalyst catalyzed the derivatization of chlorogenic acid contained in these extracts in a manner comparable to Novozym 435. Lower results were obtained for the derivatized extract from spent coffee grounds. Here, a conversion rate of 47.7% was achieved with Novozym 435 and 15.2% with the NSCG biocatalyst. It is possible that because spent coffee grounds consist mainly of chlorogenic acid, as a representative of phenolic acids, more time is needed to hydrolyze the entire amount contained in the extract. In the case of the other pomace extracts, these amounts are significantly lower.

### 2.5. Properties of Obtained Esters of Chlorogenic Acid and Pre- and Postmodified Extracts

The derivatized extracts, along with individual chlorogenic acid esters (C4-C12), were analyzed for various functional properties, including antioxidant activity, antimicrobial effects, and the ability to enhance the oxidative stability of vegetable oil. The study aimed to determine whether modifying the extracts through 1-butanol-based lipophilization could improve their overall properties, particularly in the context of developing a food additive with enhanced lipophilicity. The results for the first group of compounds—chlorogenic acid, chlorogenic acid esters, and BHT (a synthetic antioxidant used as a reference) are presented in Table 4. Among all synthesized esters, butyl chlorogenate exhibited the highest antioxidant activity (IC_50_ = 0.34 mM; TEAC = 3.26). Its performance was comparable to that of free chlorogenic acid (IC_50_ = 0.26 mM; TEAC = 3.49 and BHT (IC_50_ = 0.35 mM; TEAC = 3.14, while also displaying increased lipophilicity, as indicated by the logP value—a partition coefficient between octanol and water that reflects a molecule’s hydrophilic or hydrophobic character. It was also observed that as the carbon chain length of the alcohol used as a substrate in the esterification reaction increased, the antioxidant activity of the resulting esters decreased, regardless of the analytical method applied (CUPRAC or DPPH), despite the corresponding increase in molecular lipophilicity.

The antioxidant properties and total content of phenolic compounds in selected food waste extracts before and after lipophilization are presented in Table 5. The highest total phenolic content was observed in the spent coffee grounds extract, as confirmed by LC-MS analysis. This was followed by the chokeberry pomace extract, while the apple pomace extract exhibited the lowest concentration of phenolic compounds. A similar trend was noted in antioxidant activity, with extracts richer in phenolic content demonstrating greater antioxidant potential. A noteworthy observation concerns the impact of lipophilization on antioxidant activity. In the case of chokeberry pomace and spent coffee grounds extracts, a significant decrease in antioxidant activity was observed after lipophilization, suggesting that the process may negatively affect the overall antioxidant efficacy. This reduction could be attributed to the modification or degradation of bioactive compounds during derivatization. In contrast, only minimal changes were detected in the antioxidant activity of the apple pomace extract. Although apple pomace is considered a relatively rich source of polyphenolic compounds, over 50% of these are procyanidins, classified as flavan-3-ols. The remaining compounds include quercetin glycosides, phenolic acids, and chalcones, such as phloridzin and phloretin [41,42]. In the present study, LC-MS analysis showed that the lyophilized apple pomace extract contained the lowest total phenolic acid content (422 µg/g), compared to the chokeberry pomace (10,442 µg/g) and spent coffee grounds (15,723 µg/g) extracts.

The chlorogenic acid derivatives obtained in this study, along with extracts from food processing by-products, were also assessed for their antimicrobial properties. Eight bacterial strains, representing both Gram-positive and Gram-negative species, were tested. To each sterile disc, 10 μL of the selected compounds and extracts, prepared at a concentration of 50 μg/mL, were added. The results are summarized in Table 6 and Table 7.

Interestingly, while butyl chlorogenate exhibited antioxidant activity comparable to that of unmodified chlorogenic acid, it did not show any antimicrobial effects. Similarly, no bacterial growth inhibition was observed for BHT or chlorogenic acid. In contrast, hexyl and octyl chlorogenates demonstrated clear antimicrobial activity, particularly against Gram-positive bacteria such as *B. cereus*, *B. subtilis*, *E. faecalis*, and *S. aureus*, with the exception of *E. faecalis* in the case of octyl chlorogenate. Additionally, decyl and dodecyl chlorogenates produced smaller inhibition zones against *B. cereus*, *L. monocytogenes*, and *S. aureus*. Notably, all chlorogenic acid esters tested in this study showed selective activity only against Gram-positive bacteria, with no detectable effects on Gram-negative strains. Among the derivatives, those with medium-length alkyl chains (C6 and C8) exhibited the strongest antimicrobial activity, whereas shorter (C4) and longer chains (C10 and C12) were significantly less effective. In contrast, none of the food waste extracts exhibited antimicrobial activity against any of the tested bacterial strains.

**Figure 8:**
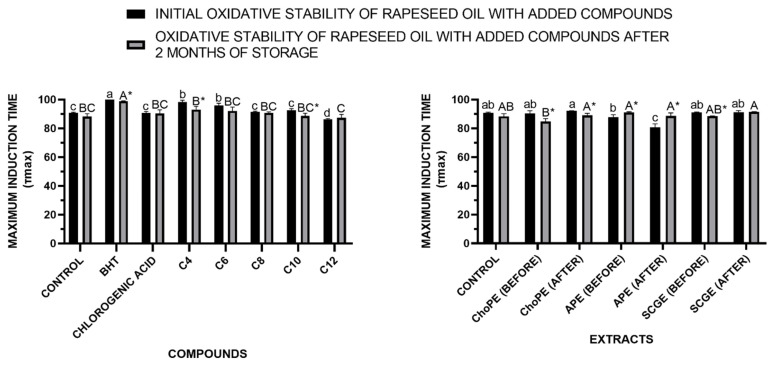
Oxidative stability of rapeseed oil with addition of chlorogenic acid (CGA), its esters with alkyl chains of different lengths (C4–C12), BHT (used as a reference antioxidant), and food waste extracts—chokeberry pomace extract (ChoPE), apple pomace extract (APE), and spent coffee grounds extract (SCGE), before and after lipophilization measured at the initial stage and after two months of storage. Values marked with the same lowercase (a–d) or uppercase (A–C) letters do not differ significantly (α = 0.05). The * symbol indicates a significant difference between the initial stadium of oxidative stability and after 2 months of storage for a given compound or extract before and after the lipophilization process. Abbreviations: ChoPE—Chokeberry Pomace Extract, APE—Apple Pomace Extract and SCGE—Spent Coffee Grounds Extract.

The oxidative stability of rapeseed oil with the addition of chlorogenic acid, its alkyl esters (C4–C12), BHT (as a reference compound), and food waste extracts was evaluated both at the initial time point and after two months of storage. As shown in Figure 8, the highest initial maximum oxidation time of vegetable oil was observed with the addition of BHT (τ_max_ = 101.8 min), followed closely by the short-chain chlorogenates, particularly the butyl (C4—τ_max_ = 98.3 min.) and hexyl (C6—τ_max_ = 95.9 min) esters. In contrast, oil with native chlorogenic acid exhibited significantly lower stability (τ_max_ = 90.7 min), which further declined after storage. This correlates with the results obtained, as the butyl and hexyl esters of chlorogenic acid exhibited the strongest antioxidant activity among the tested derivatives. Their enhanced lipophilicity relative to native chlorogenic acid likely facilitated more effective dissolution within the lipid matrix, contributing to their improved performance. After two months of storage, a slight but statistically significant reduction in the stability of oil was observed for the addition of several esters, most notably in the case of the butyl (C4—τ_max_ = 93.2 min) and decyl (C10—τ_max_ = 88.7 min) derivatives.

At the initial stage of the study, oils supplemented with SCGE (maximum oxidation time = 91.0 min.) and ChoPE (maximum oxidation time = 90.4 min) extracts, both native and lipophilized, exhibited oxidative stability levels comparable to the control oil without additives, indicating no enhancement of oxidation resistance at the start of storage. In contrast, the addition of apple pomace extract (APE), before (τ_max_ = 87.7 min) and after (τ_max_ = 80.7 min) lipohilization, led to a decrease in oxidative stability. After two months of ambient storage, oils containing all tested extracts showed similar oxidative stability levels, suggesting that while the extracts did not negatively impact stability, they also failed to provide significant protection against oxidation during prolonged storage. Overall, no significant differences in initial oxidative stability were observed among the oils with various extracts, with APE demonstrating the least effect regardless of modification. The marked decline in stability over time across most samples highlights the challenge of maintaining antioxidant efficacy throughout storage.

## 3. Discussion

### 3.1. Activity of Biocatalysts Immobilized on Food Waste Materials

Lipases are enzymes that primarily catalyze the hydrolysis of triacylglycerols into diglycerides, monoglycerides, free fatty acids, and glycerol. Beyond their hydrolytic activity, lipases also exhibit broad catalytic versatility, participating in esterification, interesterification (including acidolysis, alcoholysis, and transesterification), and aminolysis reactions. These transformations can occur in both aqueous and organic media, provided that water content is carefully regulated [15]. Given their dual ability to catalyse both hydrolysis and synthesis reactions, depending on the reaction environment, this study aimed to assess the hydrolytic and synthetic activities of selected lipase-based biocatalysts.

The high catalytic activity of the obtained immobilized preparations may be attributed to several contributing factors. Adsorption, as one of the simplest and most commonly used methods for enzyme immobilization, relies on weak physical interactions such as Van der Waals forces, hydrophobic interactions, and hydrogen bonding. Consequently, the chemical structure and surface functionality of the support material play a critical role in determining immobilization efficiency and enzyme stability. The presence of specific functional groups, such as hydroxyl, carboxyl, or phenyl moieties, on the support surface can facilitate non-covalent interactions with the enzyme, thereby enabling the formation of stable biocatalyst systems [43,44,45]. In the context of the examined agro-industrial waste materials, previous studies have suggested that fiber composition, particularly hemicellulose content, may significantly influence the adsorption capacity of the support [45,46]. Native spent coffee grounds (SCG), in comparison to apple and chokeberry pomace, exhibit a markedly higher hemicellulose content in their dry matter (Table 8). This higher polysaccharide content could enhance the density of available binding sites on the surface, thereby improving enzyme-support interactions and contributing to the stability of the immobilized system. Indeed, earlier research has demonstrated a positive correlation between hemicellulose content and the synthetic activity of biocatalysts, which is also relevant to the present study.

Moreover, the physical morphology of the support material is another key factor. Native SCGs possess a more porous structure relative to the other tested carriers, resulting in an increased specific surface area. This enhanced surface availability likely facilitates more effective adsorption of lipase molecules onto the carrier. Finally, the presence of residual lipids in raw materials may contribute to the stabilization of lipases through hydrophobic interactions that resemble the enzyme’s native environment. This is supported by the lipid content analysis of the tested waste materials, where spent coffee grounds exhibited the highest fat fraction (11%), compared to chokeberry pomace (0.3%) and apple pomace (0.2%). Lipases are characterized by polypeptide chains containing both hydrophobic and hydrophilic regions, with their active sites typically concealed by a hydrophobic lid in the closed conformation [18]. Upon contact with hydrophobic interfaces such as substrate droplets or hydrophobic supports, this lid undergoes a conformational change, exposing the active site in a process known as interfacial activation. This structural rearrangement enhances the enzyme’s catalytic efficiency and facilitates its adsorption onto hydrophobic surfaces, stabilizing the open monomeric form of the enzyme without the need for additional activation steps [46]. Furthermore, the hydrophobicity and physicochemical properties of the immobilization support significantly influence both the efficiency of lipase binding and the resulting enzymatic activity [47]. While certain lipases exhibit preferences for supports with moderate hydrophobicity, it is generally observed that more hydrophobic surfaces enable greater immobilization yields and allow for the selective fractionation of lipase mixtures based on their affinity to different supports [48]. Therefore, the higher lipid content in spent coffee grounds likely promotes enhanced lipase stabilization through such hydrophobic interactions, which is consistent with established mechanisms of lipase interfacial activation and immobilization.

### 3.2. Enzymatic Modification of Chlorogenic Acid and Food Waste Extracts

Chlorogenic acid is a widely distributed plant-derived compound, found in significant amounts in coffee beans, stone fruits, berries, and cruciferous vegetables. It is known for a range of beneficial biological effects, including strong antioxidant properties, protection of the intestinal and hepatic barriers, and demonstrated efficacy in the prevention and treatment of obesity and type II diabetes. Due to its polar nature, chlorogenic acid has limited solubility in lipid matrices, which restricts its applicability in the food, pharmaceutical, and cosmetic industries. Its hydrophobicity can be increased through chemical or enzymatic lipophilization, which involves the esterification of the carboxyl group with a fatty alcohol. The enzymatic approach is particularly favoured, as the use of biocatalysts allows for milder reaction conditions, enhanced selectivity, and minimized formation of by-products. Moreover, enzymatic reactions are environmentally friendly, requiring less energy and producing lower amounts of waste [40,49,50,51].

The optimization of the reaction parameters using the Box–Behnken design (temperature, substrate molar ratio, and enzyme concentration) revealed that the yield of chlorogenic acid esterification is especially sensitive to these factors when catalyzed by the NSCG biocatalyst. This aligns with common trends in enzymatic esterification, where increasing temperature and enzyme loading generally improve reaction efficiency, up to a point beyond which thermal deactivation or substrate saturation may occur. Conversely, the commercial immobilized biocatalyst Novozym 435 achieved high reaction yields that remained largely unaffected by variations in reaction conditions, highlighting its operational robustness and substrate tolerance. The NSCG biocatalyst was found to be more sensitive to different reaction conditions, necessitating more careful selection of parameters in experimental planning and enzyme use. In contrast, Novozym 435 performs reliably, making it a more suitable choice for large-scale and standardized production processes where stability and predictability are vital.

The enzymatic modification of chlorogenic acid using lipases has been extensively explored in prior studies, primarily using commercially available immobilized enzymes. López-Giraldo et al. [49] reported a two-step synthesis of chlorogenic acid esters: initial chemical esterification to produce methyl chlorogenate, followed by enzymatic transesterification with fatty alcohols (C4–C16) using *Candida antarctica* lipase B (CALB). The reactions, conducted at 55 °C for 96 h with varying enzyme loadings (2.5–10%, *w*/*w*), achieved conversion efficiencies ranging from 61% to 93%, depending on the alcohol chain length. Similarly, Guyot et al. [52] employed the same enzyme for direct esterification with octanol, dodecanol, and hexadecanol under milder enzyme concentrations (1.2–1.5%, *w*/*w*) and longer reaction times (30 days), achieving yields ranging from 40% to 75%. In a comparative study, Lorentz et al. [53] synthesized palmitoyl esters of chlorogenic acid using a panel of commercial lipases, including Novozym^®^ 435, Lipozyme RM-IM, TL-IM, Lipase A (*A. niger*), Lipase M (*M. javanicus*), Lipase DF (*R. oryzae*), Lipase AY (*C. rugosa*), Lipase G (*P. camembertii*), and Lipase PS (*P. cepacia*). Under standardized conditions (60 °C, 1000 rpm), bioconversion efficiency varied from 14% to 60% after 7 days, depending primarily on the substrate molar ratio of palmitic acid to chlorogenic acid (ranging from 10:1 to 80:1). Further work by Wang et al. [50] applied Lipozyme RM to acylate chlorogenic acid with vinyl esters (C2–C12) under similar thermal (55 °C) and agitation conditions (400 rpm), yielding five distinct 4-O-acylated derivative, after 7 days. Zhu et al. [54] expanded also on this approach by systematically optimizing multiple reaction parameters—including solvent type, enzyme concentration and form, substrate ratio, and time—ultimately identifying optimal conditions (55 °C, 1:10 substrate molar ratio, 400 rpm, 7 days) for efficient vinyl ester synthesis.

Despite the widespread use of immobilized commercial lipases in these studies, a significant gap remains concerning the employment of environmentally sustainable biocatalysts. To date, no reports have investigated the enzymatic lipophilization of chlorogenic acid using enzymes immobilized on biodegradable, waste-derived supports such as spent coffee grounds. This study addresses this gap by assessing the catalytic performance of a novel NSCG biocatalyst and comparing it with a commercial reference, thereby offering new insights into developing greener, cost-effective alternatives for modifying phenolic compounds. It is the first study of its kind to use microbial lipase immobilized by adsorption on spent coffee grounds to derivatize chlorogenic acid contained in food waste extract. Until now, this type of biocatalyst has only been utilized for the hydrolysis of milk fat. The immobilized lipase from *Candida rugosa* onto spent coffee grounds was used to hydrolyze bovine milk to evaluate operational stability within a real sample. This solid biocatalyst yielded nearly 60% conversion of milk fats into fatty acids after 18 h of reaction, maintaining this level across three reuses [37]. An attempt was also made to immobilize lipase from *Thermomyces lanuginosus* (TLL) onto spent coffee grounds, but during the research, it was found that it did not display any enzymatic activity and was not chosen to catalyze the synthesis of hexyl laurate [24].

This study shows that lipases are very effective in transforming specific food waste extracts. Depending on the composition of the extract, either NSCG or Novozym 435 performed better as a biocatalyst. This highlights the need to choose the right immobilized enzyme for specific reactions. The amount of product obtained can vary depending on the amount of CGA used initially and the presence of competing phenolic acids, which can block the enzyme’s active site. Other substances, such as anthocyanins, tannins, caffeine, sugars, and pectins, can also bind to or inhibit enzymes, affecting the results [24,55,56,57].

### 3.3. Properties of Obtained Esters of Chlorogenic Acid and Pre- and Postmodified Extracts

#### 3.3.1. Antioxidant Properties

When analyzing the antioxidant properties of chlorogenic acid (CGA) and its esters, a phenomenon known as “cut-off effect” or a nonlinear relationship between antioxidant activity and hydrophobicity is observed. It suggests that increasing the hydrophobicity of a polyphenolic compound does not always result in a proportional enhancement of its antioxidant capacity; on the contrary, after reaching an optimal chain length, a drastic decline in activity may occur [9,58,59,60]. Numerous studies have confirmed the nonlinear relationship between alkyl chain length and the antioxidant properties of CGA esters. One study, which included assays using the DPPH method, indicated that shorter-chain esters exhibited the highest antioxidant activity. López-Giraldo et al. [61] reported that C4 (butyl) and C8 (octyl) CGA esters exhibited significantly higher DPPH• scavenging capacity than the non-esterified 5-CQA. These esters also displayed higher reaction rate constants compared to 5-CQA. Similarly, Laguerre et al. [59], using a fibroblast model with overproduction of reactive oxygen species (ROS), observed optimal activity for the dodecyl ester (C12), with a notable 45% decrease in antioxidant capacity when the chain was extended to 16 carbon atoms, clearly illustrating the cut-off effect. Comparable results, with peak activity at C12, were also found in oil-in-water emulsions using the Conjugated Autoxidizable Triene (CAT) assay [9,62]. Conversely, a more recent study by Pappalardo et al. [63], also employing the DPPH method, reported a continuous increase in antioxidant activity with growing chain length, thus failing to confirm the classical cut-off pattern. However, it is important to note that this work referred to a general increase in activity, without a detailed analysis of a peak followed by a decline. In the context of ester synthesis, some studies [49,52] reported the highest esterification yields for C12 and C16 alkyl chain lengths. These discrepancies in optimal chain lengths (C4/C8 vs. C12) may arise from differences in the studied systems (emulsions, cellular environments, homogeneous solutions), the types of antioxidant assays, or experimental conditions.

Several mechanisms have been proposed to explain the cut-off effect. One of the most frequently cited is the partitioning hypothesis. According to this theory, esters with optimal chain lengths (e.g., butyl, octyl, dodecyl) are more effectively located at the oil–water interface in emulsions or within cellular membranes, where radical generation occurs [59,63]. In contrast, longer chains may embed too deeply in the lipid phase, thereby limiting the accessibility of hydroxyl groups to reactive species [63,64]. For example, dodecyl chlorogenate exhibited a greater partitioning capacity into the oil phase compared to longer esters (C16, C18, C20), which is crucial for optimal activity [59]. A second explanation involves steric effects: bulky alkyl chains may hinder interactions with free radicals by restricting access to reactive sites. López-Giraldo et al. [61] suggested that steric hindrance may also limit dimer formation in longer esters (e.g., dodecyl) compared to shorter ones (e.g., butyl), thus affecting overall antioxidant potential. A third proposed mechanism relates to self-assembly behaviour. In cellular studies, the superior activity of dodecyl chlorogenate may be associated with its ability to self-organize in aqueous environments, forming structures that better interact with cell membranes [59].

Accordingly, the effects of lipophilization may have been more pronounced in the latter two by-products due to their higher content of phenolic acids, which are more susceptible to chemical modification. Lipophilization of whole extracts carries the inherent risk that the synergistic interactions among phenolic constituents may be altered, potentially diminishing or enhancing antioxidant properties. In this study, butyl chlorogenate exhibited the highest antioxidant activity among the derivatives tested. However, the antioxidant potential of other phenolic acid derivatives formed during the process remains unknown, and their identification and evaluation warrant further investigation.

#### 3.3.2. Antimicrobial Properties

The obtained results are partially consistent with those of other studies. Suárez-Quiroz et al. [65] evaluated both native chlorogenic acid (CGA) and its dodecyl ester (DCGA) derived from green coffee for their antimicrobial potential. While DCGA was active against select Gram-positive bacteria (*B. cereus*, *Clostridium sporogenes*, *Listeria innocua*), it showed no activity against Gram-negative strains such as *E. coli*, *Pseudomonas fluorescens*, and *Salmonella enterica*. Interestingly, unmodified CGA exhibited a broader antimicrobial spectrum, including activity against both Gram-positive and Gram-negative bacteria such as *P. fluorescens* and *S. aureus*, although *S. enterica* was resistant to both forms. The authors suggested that lipophilization through esterification may enhance antimicrobial efficacy in certain contexts, although it has a narrower spectrum of activity in others.

Further support for the antimicrobial potential of CGA derivatives comes from Ma et al. [66], who synthesized chlorogenic acid analogues with lipophilic chains containing amino acid residues. Two of these compounds, featuring threonine moieties, showed significantly enhanced antifungal activity, particularly against drug-resistant *Candida krusei*, while also demonstrating reduced toxicity toward marine organisms.

These findings collectively support the notion that CGA and its derivatives possess notable, although structure-dependent, antimicrobial properties. In light of the current results, it appears that the antimicrobial efficacy of chlorogenic acid derivatives is strongly influenced by the length and nature of the alkyl chain introduced during the esterification process. Medium-chain esters such as hexyl and octyl chlorogenates offer the most promising activity, particularly against Gram-positive pathogens, and may serve as potential candidates for further development as natural antimicrobial agents. However, their selectivity and lack of effect on Gram-negative bacteria highlight the need for continued structural optimization to broaden their spectrum of activity.

#### 3.3.3. Oxidative Stability in Rapeseed Oil

Despite the high antioxidant activity of the extracts themselves, particularly those derived from spent coffee grounds and chokeberry pomace, this effect was not reflected in improved oxidative stability of the oil. It is possible that derivatization of the food waste extracts with 1-butanol was insufficient to ensure adequate solubility in the oil matrix, and the use of alcohols with longer alkyl chains may be required in future studies. For instance, in the study by Aladedunye et al. [67], a polyphenolic extract from rowanberry (*Sorbus aucuparia*) was developed with the aim of enriching oil with the obtained compound. The incorporation of an octadecyl chain significantly enhanced the antioxidant performance of chlorogenic acid in rapeseed oil during deep-fat frying of French fries; however, it had no considerable effect on the oxidative stability of the oil under storage conditions. Compared to the native compound, the lipophilized phenolic derivative showed better transfer into the fried product, potentially improving the functional properties, nutritional quality, and shelf life of the final food product. Similarly, in the study by Aladedunye and Matthäus [68], a native phenolic extract from Canadian crabapple, composed primarily of phloridzin, was enzymatically modified to obtain an octadecanoyl derivative. The introduction of an octadecyl chain significantly improved the antioxidant capacity of phloridzin in rapeseed oil during deep frying, although no significant effect was observed on oxidative stability during storage. The addition of the modified phenolic extract to frying oil substantially improved tocopherol retention in fried foods, thereby enhancing their nutritional value and potentially extending their shelf life during storage.

## 4. Materials and Methods

### 4.1. Materials

In the present research, the liquid lipase of microbial origin—Novozym 51032 from *Aspergillus oryzae* (current trade name Sustine 140)—was kindly provided by Novozymes (Bagsvaerd, Denmark) and used as a biocatalyst. The food waste materials, which were carriers for the enzyme immobilization process, were acquired from the coffee shops—spent coffee grounds and juices production—apple and chokeberry pomaces (Greenherb Company, Wysoka, Poland). The biocatalyst Novozym 435 was used as a reference. All chemical reagents and solvents were purchased from Sigma-Aldrich (Poznań, Poland).

### 4.2. Preparation of Food Waste

**Figure 9:**
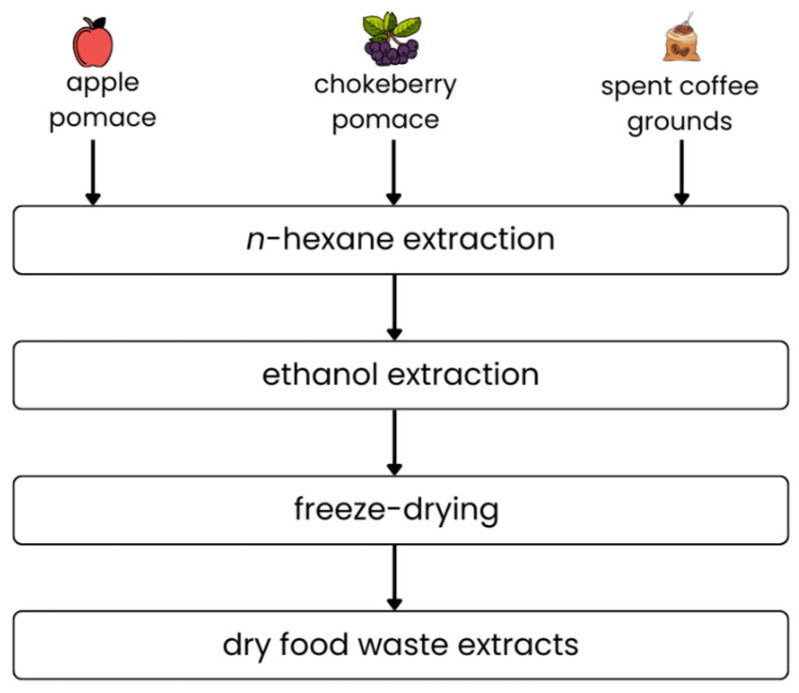
The scheme of the food waste material pretreatment process.

A short diagram of the extract pre-treatment process is shown in Figure 9. Ten grams of each food waste material was weighed, wrapped in filter paper, and placed in a Soxhlet apparatus. Initially, extraction was performed using 150 mL of *n*-hexane, followed by 150 mL of ethanol. For each step, the extract was poured for 10 cycles of solvent transfer. After that time, the residual solvent was evaporated. In the case of hexane extracts, the solvent was evaporated, and the lipid contents in the food waste were calculated. The amount of fat extracted was calculated by relating the weight of the extracted lipids to the volume of food waste used in the process. The obtained ethanol extracts were put on the plates and frozen at −42 °C in an Irinox freezer (Corbanese, Italy) for 1 h and then moved for 24 h to the lyophilization process in the Christ Gamma 1–16 LSC apparatus (Osterode am Harz, Germany). The applied conditions were: a temperature of 10 °C on the shelves and a pressure of 63 Pa. The safety pressure was 103 Pa, protecting the material from temperatures above −20 °C. The dry extracts were stored at room temperature.

### 4.3. Lipase Immobilization Procedure

Liquid lipase from *A. oryzae* was immobilized based on the procedure described in the study by Jasińska et al. [69]. Briefly, 1 g of food waste (native spent coffee grounds—SCG; apple pomace—AP; or chokeberry pomace—ChoP) was added to a flask. Next, 1 mL of liquid lipase and 14 mL of distilled water were added to the flask. The lipase solution and the food waste carrier were agitated together for 2 h using a magnetic stirrer (140 rpm). After this period, the resulting biocatalysts were filtered, washed with distilled water, and dried at room temperature. The immobilization reactions were performed in triplicate for each type of food waste material.

### 4.4. Lipase Activity Assay—Hydrolytic and Synthetic Activities

To determine the hydrolytic and synthetic activities of the immobilized enzyme preparations, two methods were employed as detailed in the research by Jasińska et al. [69]. Both methods relied on spectrophotometric measurements. Based on the results of the lipolytic activities, a decision was made to select the most active biocatalyst for further testing.

The first method focused on the hydrolysis reaction of *p*-nitrophenyl laurate, which was conducted for 15 min at 37 °C. In this procedure, 25 mg of the immobilized enzyme preparation was combined with 100 µL of distilled water and 25 µL of *p*-nitrophenyl laurate solution (0.3 mmol dissolved in 2 mL of heptane) in Eppendorf test tubes. The mixture was stirred, and the absorbance was measured at 410 nm using a UV-Vis spectrophotometer. The unit of lipase enzymatic activity was defined as 1 U, which is the amount of enzyme that releases 1 µmol of *p*-nitrophenol per minute under the specified assay conditions.

The second method was based on the transesterification reaction, which occurred in an Eppendorf tube containing 100 mM vinyl acetate and 100 mM 1-butanol in 1 mL of *n*-hexane. To this mixture, 5 mg of immobilized lipase was added. After incubating for 5 min, diluted samples were prepared in test tubes. To each sample, 1 mL of a 0.1% (*m*/*v*) solution of MBTH (3-methyl-2-benzothiazolinone hydrazone hydrochloride hydrate) was added and agitated for 10 min at 30 °C. Following this, 0.4 mL of a 1% (*m*/*v*) solution of NH_4_Fe(SO_4_)_2_ 12H_2_O (dissolved in 0.1M HCl) was added, and the mixture was mixed for 30 min at 30 °C. Spectrophotometric measurements were then carried out at 595 nm.

### 4.5. Elemental Compositions of Biocatalysts and Their Supports

Total C, N, and S contents of three biocatalysts and their supports were determined by dry combustion (Vario MacroCube, Elementar, Langenselbold, Germany).

### 4.6. Optimization of Synthesis Reaction—Box–Behnken Design

**Figure 10:**
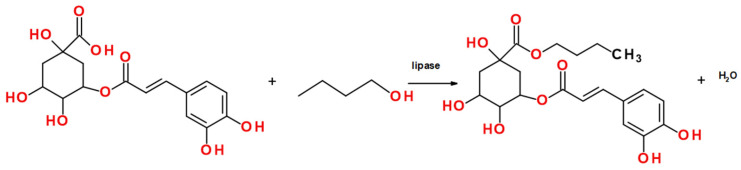
Reaction scheme for the synthesis of chlorogenic acid with 1-butanol.

A model reaction was performed for the lipophilization of chlorogenic acid with 1-butanol (Figure 10) using biocatalyst—lipase from *A. oryzae* immobilized onto spent coffee grounds (NSCG). The following three-factor, three-level experiment was designed using Box–Behnken methodologies to optimize the reaction conditions (Table 9). The analyzed factors included temperature (35, 45, and 55 °C), enzyme concentration (20%, 35%, and 50%), and substrate molar ratio (alcohol to chlorogenic acid—2:1, 5:1, and 8:1). The tert-butyl methyl ether was used as a solvent (15 mL). The time duration of the reaction was also optimized (3, 5, 7, and 9 days). The subsequent reactions involved the lipophilization of chlorogenic acid with alcohols of varying carbon chain lengths (C6, C8, C10, C12), which were carried out over 7 days. The optimized parameters are a part of the patent application “Method for the preparation of chlorogenic acid esters and alcohols, by biocatalysis. WIPO ST 10/C PL451973“. Experiments were conducted using two biocatalysts: NSCG and Novozym 435, which served as a reference. The reaction yield was calculated from the area under the substrate and product peaks on the chromatogram obtained using the HPLC technique.

### 4.7. Chromatographic Determination of Esterified Post-Reaction Mixtures

The process for preparing esterified post-reaction mixtures involved evaporating the reaction solvent under nitrogen, diluting it with methanol, and then transferring the mixture to 25 mL volumetric flasks. Subsequently, the prepared solutions of esterified post-reaction mixtures were filtered using a PTFE syringe filter (0.45 µm) into chromatographic vials.

Chromatographic analysis of chlorogenic acid esters was performed using a modified analytical procedure reported by Głowacka et al. [70]. HPLC analysis was conducted by means of the high-performance liquid chromatography system from Shimadzu (Kyoto, Japan), which consisted of a DGU-20A SR degassing unit, an LC-20AD pump, a SIL-20A HT autosampler, a CTO-10AS VP column oven, and a SPD-M20A diode array detector. The obtained samples were separated on the Supelco 5-µm SUPELCOSIL LC-18-S analytical column (25 cm × 4.6 mm). The chromatographic separation of the obtained samples was performed in gradient elution mode using a 0.1% aqueous formic acid solution (phase A) and methanol (phase B) as mobile phases, at a flow rate of 0.8 mL/min. The composition of the mobile phase was as follows: 0 min, 80% B; 12–15 min, 50% B; 28–45 min, 80% B. Data were registered at a wavelength of 325 nm. In order to determine the degree of reaction, the areas under the peaks of the analytes of interest were utilized.

### 4.8. Column Chromatography and NMR Analysis

Upon completion of the reaction, the mixture was filtered to separate the biocatalyst. The solvent was then evaporated under reduced pressure, and the resulting residue was subjected to column chromatography for the purification of chlorogenic acid esters. Silica gel 60 (particle size: 0.040–0.063 mm; mesh size: 230–400) was used as the stationary phase, while a chloroform: methanol mixture (1:1, *v*/*v*) served as the mobile phase. The presence of target compounds was monitored by thin-layer chromatography (TLC) using silica gel plates.

Fractions containing the desired product were pooled, the solvent was removed by evaporation, and the esters were crystallized from heptane to obtain a purified compound. Structural confirmation of the purified esters was performed by carbon-13 nuclear magnetic resonance spectroscopy (^13^C NMR). Spectra were recorded using a Bruker AVANCE 500 MHz spectrometer (Bruker, Billerica, MA, USA) with DMSO-d6 as the solvent. Chemical shifts (δ) are reported in parts per million (ppm) and referenced to tetramethylsilane (TMS) as the internal standard.

Butyl chlorogenate
^13^C NMR (126 MHz, DMSO-d6) δ 173.19, 165.43, 148.61, 145.70, 145.17, 125.34, 121.34, 115.85, 114.55, 113.81, 73.12, 71.09, 69.40, 66.91, 64.12, 37.23, 35.09, 30.03, 18.54, 13.56.
Hexyl chlorogenate
^13^C NMR (126 MHz, DMSO-d6) δ 173.16, 165.35, 148.54, 145.66, 145.15, 125.32, 121.27, 115.79, 114.55, 113.75, 73.01, 71.07, 69.27, 66.75, 64.37, 37.22, 34.97, 30.80, 27.88, 24.90, 21.97, 13.78.
Octyl chlorogenate
^13^C NMR (126 MHz, DMSO-d6) δ 173.16, 165.35, 148.53, 145.65, 145.15, 125.31, 121.26, 115.77, 114.53, 113.73, 73.01, 71.07, 69.28, 66.77, 64.36, 37.22, 34.99, 31.11, 28.57, 27.92, 25.24, 22.05, 13.94.
Decyl chlorogenate
^13^C NMR (126 MHz, DMSO-d6) δ 173.16, 165.36, 148.57, 145.67, 145.16, 125.29, 121.27, 115.77, 114.51, 113.71, 73.04, 71.06, 69.29, 66.80, 64.37, 37.22, 35.03, 31.31, 29.12, 29.00, 28.97, 28.92, 28.85, 28.73, 22.10, 13.97.
Dodecyl chlorogenate
^13^C NMR (126 MHz, DMSO-d6) δ 173.15, 165.35, 148.54, 145.65, 145.14, 125.30, 121.25, 115.76, 114.51, 113.73, 73.04, 71.05, 69.31, 66.80, 64.35, 37.21, 35.03, 31.30, 29.02, 28.99, 28.91, 28.89, 28.72, 28.60, 27.92, 25.24, 22.11, 13.97.

### 4.9. ESI- MS Experiments

High-resolution ESI-MS spectra of butyl chlorogenate were acquired using an ultra-performance liquid chromatograph ACQUITY UPLC I-Class (Waters, Milford, MA, USA) coupled with a Synapt G2-S HDMS (Waters, Milford, MA, USA) mass spectrometer equipped with an electrospray ion source and q-TOF type mass analyser. The instrument was controlled, and the recorded data were processed using the MassLynx V4.1 software package (Waters, Milford, MA, USA). The electrospray ionization–mass spectrometry (ESI–MS) spectra were recorded in the positive and negative ion mode in the m/z range 50–3000.

### 4.10. Lipophilization Process

Based on the optimization of the synthesis of chlorogenic acid derivatives, parameters were selected to carry out the lipophilization of extracts. Reactions were maintained in conical flasks at 55 °C, stirring continuously (150 rpm). Into the reaction mixture, 1-butanol and dried extracts from food waste were added in a 8:1 (*w*/*w*) ratio, along with 15 mL of tert-butyl methyl ether. The reaction was catalyzed by immobilized onto spent coffee grounds lipase from *A. oryzae* and Novozym 435, as a reference, for 7 days. The biocatalysts were removed from the reaction mixture by filtration. The extracts, after evaporating the remaining solvent, were analyzed by chromatographic techniques.

### 4.11. LC-MS Assay of the Obtained Extracts

The comparison of the content of selected phenolic acids and their derivatives in prepared extracts before and after the lipophilization process was determined by Liquid Chromatography-Mass Spectrometry.

Each lyophilized extract was accurately weighed (10 mg) and dissolved in 1 mL of methanol. The suspensions were sonicated in an ultrasonic bath for 30 min at room temperature. Subsequently, the samples were filtered through 0.22 µm syringe filters (PTFE membrane). Based on these stock solutions, three concentration levels of analytes were prepared: 10, 1, and 0.1 mg/mL. To achieve this, the stock solutions were evaporated to dryness under a stream of nitrogen at room temperature, and the residues were then dissolved in a 30% methanol solution (*v*/*v*) to obtain the desired concentrations. The contents of the compounds were expressed on a dry weight basis (per gram of dry extract).

To determine the content of phenolic acids in the derivatized extracts, aliquots were taken from the reaction mixture and processed to dryness under a stream of nitrogen. The residues were reconstituted in 30% methanol (*v*/*v*), and the final volume was adjusted to ensure comparability between samples. In most cases, this meant restoring the same concentration as in the original extract; in some instances, the volume was increased to introduce an additional dilution when required by the limited amount of material available. The prepared samples were then subjected to LC-MS analysis. The content of compounds was expressed per liter of derivatized extracts.

### 4.12. Total Polyphenol Content

The total polyphenolic content in extracts (1 mg/mL in methanol) before the lipophilization process was determined using the Folin–Ciocalteu method. 0.18 mL of extract was placed in glass tubes and diluted with 4.92 mL of distilled water. Afterwards, 0.3 mL of Folin–Ciocalteu reagent was added and mixed. After 3 min, the pH of the solution was adjusted by adding and mixing 0.6 mL of a supersaturated sodium carbonate solution. The incubation was carried out in the darkness at 25 °C for an hour. The absorbance of the solutions was measured using a Rayleigh UV-1601 spectrophotometer (BRAIC, Beijing, China) at a wavelength of 750 nm against a blank sample. The content of polyphenols was calculated as chlorogenic acid equivalents (mg CGA equivalents/ g extract). The analysis was conducted in triplicate.

### 4.13. Evaluation of Antioxidant Properties

#### 4.13.1. The DPPH Assay

The DPPH assay was performed according to the protocol outlined by Zanetti et al. [71], with some minor adjustments made to assess the antioxidant properties of the obtained compounds and extracts. Briefly, 0.004% solutions of DPPH, 1 mM of isolated chlorogenic esters, and 1 mg/mL of the obtained extracts in methanol were prepared. The antioxidant activities of the tested compounds were measured using a Rayleigh UV-1601 spectrophotometer (BRAIC, Beijing, China) at 517 nm. Based on the results of four different dilutions of tested solutions, the IC_50_ parameters, i.e., the concentration required for a 50% reduction of the DPPH· radical, were calculated. Butylated hydroxytoluene (BHT) was used as a reference.

#### 4.13.2. CUPRAC Method

The CUPRAC (cupric ion reducing antioxidant capacity) assay was utilized as a supplementary method to evaluate the antioxidant activities of the substances under investigation. This analysis was conducted following the approach outlined by Özyürek et al. [72]. In this method, the absorbance of the complex formed between neocuproine (2,9-dimethyl-1,10-phenanthroline) and the Cu(I) ion is assessed spectrophotometrically at 450 nm, with antioxidant compounds acting as electron donors. The Trolox Equivalent Antioxidant Capacities (TEAC) were calculated for the chlorogenic esters and extracts being tested based on the absorbance readings of these compounds compared to Trolox, which served as a standard reference. Butylated hydroxytoluene (BHT) was used as a reference.

### 4.14. Evaluation of Antimicrobial Properties

The antimicrobial activity was evaluated using the disc diffusion assay. Esters and extracts were first dissolved in ethanol to achieve a concentration of 50 mg/mL. Then, 10 μL of each compound and extracts were applied onto sterile 6 mm diameter discs. Bacterial suspensions adjusted to a 0.5 McFarland standard (1.5 × 10^8^ CFU/mL) were evenly spread onto Mueller–Hinton agar plates. The discs impregnated with the selected esters and extracts were then placed onto the inoculated agar surface. Plates were incubated at 37 °C for 16–18 h. After incubation, the diameters of the inhibition zones around the discs were measured to assess antibacterial efficacy. The following bacteria, purchased from the Polish Collection of Microorganisms (PCM) of the Institute of Immunology and Experimental Therapy, Polish Academy of Sciences (Wrocław, Poland), were used for analysis: *Bacillus cereus* PCM 482, *Bacillus subtilis* PCM 486, *Enterobacter cloacae* PCM 2848, *Enterococcus faecalis* PCM 2909, *Escherichia coli* PCM 2057, *Listeria monocytogenes* PCM 2191, *Serratia marcescens* PCM 549, and *Staphylococcus aureus* PCM 2054.

### 4.15. PDSC Measurements

The maximum oxidation time (τ_max_) for rapeseed oils without (control sample) and with additives was determined using a pressure differential scanning calorimetry (PDSC) assay. Into 30 g of rapeseed oil in Falcon tubes, the ethanolic extracts were added (3 mg of compound/extract in 30 µL EtOH), resulting in a 0.01% concentration of the compound/extract in oil. The measurement was conducted immediately after the additives were applied and after a 2-month storage period had elapsed. An oil sample of 3–4 mg was placed in an open aluminum pan, with an empty reference pan next to it, within the pressure chamber of a DSC Q20 apparatus (TA Instruments, New Castle, DE, USA). The τ_max_ was recorded at a constant temperature of 120 °C and an initial pressure of 1400 kPa, all within a pure oxygen environment. The τ_max_, measured in minutes, was derived by analyzing the heat flow over time with the help of TA Software (version 4.5A). All tested compounds were added at a concentration of 0.01% (m/m) in alignment with the maximum BHT addition to fats and oils designated for the production of heat-treated foods or frying oils and fats, in accordance with EU Regulation [73].

### 4.16. Lipophilicity Analysis

To determine the lipophilicity of the obtained chlorogenic acid esters, the octanol/water partition coefficient (logP) and aqueous solubility (logS) were calculated in MOE Software (MOE 2015.10, Molecular Operating Environment, Chemical Computing Group, Montreal, QC, Canada).

### 4.17. Statistical Analysis

The results were statistically analyzed using the STATISTICA 13.3 software (TIBCO Software Inc., Palo Alto, CA, USA). The following methods were applied: the Shapiro–Wilk test to assess the statistical hypothesis regarding the normality of the distribution of the experimental data, Levene’s and Brown-Forsythe tests to evaluate the hypothesis of variance homogeneity, analysis of variance (ANOVA), post-hoc Tukey’s test, and the Box–Behnken design for optimizing the synthesis reaction. A significance level of *p* ≤ 0.05 was deemed statistically significant.

## 5. Conclusions

This study successfully demonstrates the dual potential of plant-based food processing by-products from beverage production as valuable sources of phenolic acids and sustainable supports for lipase immobilization. Microbial lipase from *A. oryzae* was successfully immobilized on three biodegradable carriers: spent coffee grounds (NSCG), chokeberry pomace (NChoP), and apple pomace (NAP). Among these, the biocatalyst immobilized on native spent coffee grounds (NSCG) exhibited the highest enzymatic activity, substantially surpassing the other preparations. This enhanced performance of NSCG may be attributed to its higher hemicellulose content, more porous structure, and elevated lipid fraction (11%), which favour interfacial activation of lipase and stabilization of the enzyme through hydrophobic interactions. Chlorogenic acid (CGA), abundant in all extracts, was enzymatically lipophilized to enhance its compatibility with lipid matrices. The lipophilization yield increased with the length of the alcohol’s carbon chain using the biocatalyst—NSCG, while the commercial enzyme showed consistent performance regardless of chain length. This is the first report of using a food waste-immobilized biocatalyst for chlorogenic acid lipophilization.

Characterization of the synthesized derivatives and modified extracts revealed diverse effects of structural modification. Butyl chlorogenate exhibited the highest antioxidant activity among all synthesized esters, comparable to that of free CGA and BHT, while also offering improved lipophilicity. A clear “cut-off effect” was observed, where longer alkyl chains led to decreased antioxidant activity, likely due to suboptimal orientation at the lipid–water interface or steric hindrance. Regarding antimicrobial activity, medium-chain chlorogenates (C6 and C8) showed the strongest antibacterial effects, particularly against Gram-positive bacteria. In contrast, free CGA, BHT, butyl chlorogenate (C4), and longer esters (C10 and C12) did not exhibit such activity. For the extracts, lipophilization of chokeberry pomace and spent coffee grounds extracts resulted in a significant decrease in antioxidant activity, suggesting that the process may have disrupted the stability of active components. Importantly, none of the tested extracts, either native or lipophilized, demonstrated antibacterial activity. In terms of oxidative stability of rapeseed oil, short-chain chlorogenates (C4 and C6) exhibited the highest initial stability, likely due to better solubility in the lipid matrix compared to free CGA. However, the extracts themselves, even those rich in polyphenols, did not significantly improve the oxidative stability of the oil. This indicates the need for further research to optimize their solubility in lipid environments, possibly through the use of alcohols with longer alkyl chains during the lipophilization process.

In conclusion, food processing by-products represent a promising, environmentally friendly, and sustainable material for enzyme immobilization in biocatalytic processes, enabling the production of valuable bioactive compounds. While the yield and functionality of the resulting derivatives depend on extract composition and alkyl chain length, this study opens new perspectives for utilizing food waste as a raw material for the development of innovative food additives with enhanced lipophilicity and potential antioxidant and antibacterial properties. Further investigations should focus on detailed identification of the derivatives formed during extract lipophilization and on optimizing reaction conditions to maximize desired properties for specific applications.

## 6. Patents

Karina Jasińska, Agata Fabiszewska and Bartłomiej Zieniuk have a patent application: “Method for the preparation of chlorogenic acid esters and alcohols, by biocatalysis.” WIPO ST 10/C PL451973.

## Figures and Tables

**Table 1:** Carbon, nitrogen and sulfur content of three supports and three immobilized lipases. The * symbol indicates a significant difference between the results for each support and biocatalyst, which is immobilized on this carrier.

Source	Percentage Content (%)		
	C	N	S
Spent coffee grounds (SCG)	48.41 ± 0.01	2.23 ± 0.01	0.12 ± 0.00
Novozym 51032^®^ immobilized on spent coffee grounds (NSCG)	50.07 ± 0.02 *	2.37 ± 0.00 *	0.12 ± 0.00
Chokeberry pomace (ChoP)	50.31 ± 0.01	1.87 ± 0.02	0.09 ± 0.00
Novozym 51032^®^ immobilized on chokeberry pomace (NChoP)	50.49 ± 0.42	2.17 ± 0.01 *	0.10 ± 0.00 *
Apple pomace (AP)	44.62 ± 0.06	0.99 ± 0.02	0.07 ± 0.00
Novozym 51032^®^ immobilised on apple pomace (NAP)	44.65 ± 0.09	1.26 ± 0.01 *	0.07 ± 0.00

**Table 2:** Experimental matrix for the three-variable Box–Behnken design with the yield of the butyl chlorogenate synthesis catalyzed by lipase from *A. oryzae* immobilized onto spent coffee grounds (NSCG) and Novozym 435.

Exp. No.	Temperature (°C)	Substrate Molar Ratio (Alcohol: Chlorogenic Acid)	Enzyme Concentration Relative to the Total Mass of Substrates (%)	Yield (%) NSCG	Yield (%) Novozym 435
1	35	2:1	35	1.78	17.73
2	55	2:1	35	1.67	12.81
3	35	8:1	35	9.24	49.12
4	55	8:1	35	16.02	95.67
5	35	5:1	20	2.40	30.61
6	55	5:1	20	0.96	39.21
7	35	5:1	50	10.11	42.32
8	55	5:1	50	9.39	66.42
9	45	2:1	20	1.91	8.45
10	45	8:1	20	7.20	57.10
11	45	2:1	50	5.00	31.72
12	45	8:1	50	15.91	59.32
13	45	5:1	35	10.94	52.81
14	45	5:1	35	10.88	40.81
15	45	5:1	35	10.82	42.11

**Table 3:** Conversion rate of butyl chlorogenate in the reaction mixture after lipophilization of food waste extracts, catalyzed by Novozym 435 and NSCG.

Conversion Rate of Butyl Chlorogenate in Extracts After Lipophilization (%)					
Chokeberry Pomace Extract		Apple Pomace Extract		Spent Coffee Grounds Extract	
Novozym 435	NSCG	Novozym 435	NSCG	Novozym 435	NSCG
90.9%	92.0%	91.5%	87.6%	47.7%	15.2%

**Table 4:** Selected properties—antioxidant activity by DPPH and CUPRAC methods, and lipophilicity of chlorogenic acid and its esters, and BHT. The values with the same lowercase letter (a–d) within the method did not differ significantly (α = 0.05).

Compound	Antioxidant Properties		Lipophilicity	
	CUPRAC— TEAC	DPPH— IC_50_ (mM)	logP	logS
BHT	3.14 ± 0.22 ^a^	0.35 ± 0.02 ^a^ ^b^	4.964	−4.909
Chlorogenic acid	3.49 ± 0.32 ^a^	0.26 ± 0.00 ^a^	0.415	−1.489
Butyl chlorogenate	3.26 ± 0.17 ^a^	0.34 ± 0.00 ^a^ ^b^	2.076	−2.945
Hexyl chlorogenate	1.15 ± 0.15 ^b^	0.40 ± 0.01 ^b^	2.960	−3.976
Octyl chlorogenate	0.80 ± 0.05 ^b^ ^c^	0.58 ± 0.01 ^c^	3.844	−5.007
Decyl chlorogenate	0.64 ± 0.03 ^c^	0.57 ± 0.01 ^c^	4.728	−6.037
Dodecyl chlorogenate	0.45 ± 0.02 ^c^	0.91 ± 0.12 ^d^	5.612	−7.067

**Table 5:** Antioxidant properties and total content of phenolic compounds in selected food waste extracts before and after lipophilization process. Means with the same lowercase letter (a–c) or capital letter (A–C) within the method did not differ significantly (α = 0.05). The * symbol indicates a significant difference between the results for a given extract before and after the lipophilization process. Abbreviation: n.a.—not analyzed.

Type of Extract	Lipophilization	Antioxidant Properties		Total Content of Phenolic Compounds
		CUPRAC— TEAC	DPPH— IC_50_ (mg/mL)	Average CGA Content (mg/mL)
Chokeberry pomace extract	BEFORE	2.00 ± 0.17 ^b^	0.26 ± 0.02 ^a^	0.26 ± 0.03 ^b^
	AFTER	0.85 ± 0.07 ^B^ *	0.90 ± 0.02 ^B^ *	n.a.
Apple pomace extract	BEFORE	0.23 ± 0.01 ^c^	4.55 ± 5.97 ^b^	0.03 ± 0.01 ^c^
	AFTER	0.23 ± 0.01 ^C^	5.97 ± 0.02 ^C^ *	n.a.
Spent coffee grounds extract	BEFORE	2.50 ± 0.10 ^a^	0.13 ± 0.09 ^a^	0.36 ± 0.00 ^a^
	AFTER	1.60 ± 0.07 ^A^ *	0.42 ± 0.04 ^A^ *	n.a.

**Table 6:** Comparison of the antimicrobial activity of chlorogenic acid, its esters and BHT as a reference. The 6 mm indicates no growth inhibition. *—A diameter of an inhibition zone of 6 mm is considered as no antimicrobial activity.

Compound	Inhibition Zone Diameter (mm)							
	*B.* *cereus* PCM 482	*B.* *subtilis* PCM 486	*E.* *faecalis* PCM 2909	*L.* *monocytogenes* PCM 2191	*S.* *aureus* PCM 2054	*E.* *cloacae* PCM 2848	*E.* *coli* PCM 2057	*S.* *marcescens* PCM 549
BHT	6 *	6	6	6	6	6	6	6
Chlorogenic acid	6	6	6	6	6	6	6	6
Butyl chlorogenate	6	6	6	6	6	6	6	6
Hexyl chlorogenate	10	8	10	6	12	6	6	6
Octyl chlorogenate	10	8	6	6	10	6	6	6
Decyl chlorogenate	8	6	6	9	9	6	6	6
Dodecyl chlorogenate	7	6	6	7	6	6	6	6

**Table 7:** Comparison of the antimicrobial activity of spent coffee grounds, chokeberry and apple pomace extracts before and after lipophilization. The 6 mm indicates no growth inhibition. *—A diameter of an inhibition zone of 6 mm is considered as no antimicrobial activity.

Type of Extract	Lipophilization	Inhibition Zone Diameter (mm)							
		*B.* *cereus* PCM 482	*B.* *subtilis* PCM 486	*E.* *faecalis* PCM 2909	*L.* *monocytogenes* PCM 2191	*S.* *aureus* PCM 2054	*E.* *cloacae* PCM 2848	*E.* *coli* PCM 2057	*S.* *marcescens* PCM 549
Chokeberry pomace extract	BEFORE	6 *	6	6	6	6	6	6	6
	AFTER	6	6	6	6	6	6	6	6
Apple pomace extract	BEFORE	6	6	6	6	6	6	6	6
	AFTER	6	6	6	6	6	6	6	6
Spent coffee grounds extract	BEFORE	6	6	6	6	6	6	6	6
	AFTER	6	6	6	6	6	6	6	6

**Table 8:** Cellulose, hemicellulose, and lignin content of supports—native apple and chokeberry pomaces and spent coffee grounds. Abbreviations: ADF—acid detergent fiber, ADL—acid detergent lignin, NDF—neutral detergent fiber, DM—dry mass.

Food Waste	Unit	Cellulose (ADF-ADL)	Hemicellulose (NDF-ADF)	Lignin (ADL)	
Native Apple Pomace	%DM	20.99 ± 0.07	5.87 ± 0.97	9.46 ± 0.20	[35]
Native Chokeberry Pomace		18.87 ± 0.32	3.53 ± 1.30	32.76 ± 0.08	[35]
Native Spent Coffee Grounds		21.06 ± 0.41	23.69 ± 0.97	16.87 ± 0.48	[36]

**Table 9:** Coded levels and decoded value of the Box–Behnken design.

Factors	Name	Units	Low (−1)	Medium (0)	High (+1)
1	Temperature	°C	35	45	55
2	Substrate molar ratio	Alcohol:chlorogenic acid	2:1	5:1	8:1
3	Enzyme concentration	%	20	35	50

## Data Availability

The raw data supporting the conclusions of this article will be made available by the authors on request.

## Abbreviations

The following abbreviations are used in this manuscript:

AP	Apple pomace
NAP	Lipase from *A.oryzae* (Novozym 51032) immobilized on apple pomace
CGA	Chlorogenic acid
ChoP	Chokeberry pomace
NChoP	Lipase from *A.oryzae* (Novozym 51032) immobilized on chokeberry pomace
NSCG	Lipase from *A.oryzae* (Novozym 51032) immobilized on spent coffee grounds
SCG	Spent coffee grounds
